# Cross-Canada Release of the Post-Secondary Student Stressors Index (PSSI): Protocol for a Cross-sectional, Repeated Measures Study

**DOI:** 10.2196/27713

**Published:** 2021-08-31

**Authors:** Brooke Linden

**Affiliations:** 1 Health Services and Policy Research Institute Queen's University Kingston, ON Canada

**Keywords:** stress, mental health, health promotion, postsecondary, higher education, measurement tool, study protocol

## Abstract

**Background:**

The prevalence of mental health–related problems, including stress, psychological distress, and symptoms of mental illnesses, continues to increase among Canadian postsecondary student populations. Excessive stress in this population has been linked to a number of negative academic and health outcomes. Despite attempts to improve mental health at postsecondary institutions, a persistent gap exists in the evaluation of the specific sources of stress for students within the postsecondary setting.

**Objective:**

The purpose of this paper is to report the study protocol for a cross-Canada, multisite launch of the Post-Secondary Student Stressors Index (PSSI), which will engage postsecondary institutions across the country as partners and facilitate improved measurement of the sources of student stress, in addition to contributing toward improved tailoring of upstream mental health services and support.

**Methods:**

Created in collaboration with students, the PSSI is a validated 46-item tool assessing stressors across five domains: academics, learning environment, campus culture, interpersonal, and personal stressors. The tool is designed to be applicable to students at all years, levels, and areas of study. Data will be collected longitudinally at multiple time points over the course of each academic year.

**Results:**

We recruited 15 postsecondary institutions across Canada for the first year, inviting students to participate in an online survey including questions concerning sociodemographic characteristics, stress, mental health, and resiliency. Analyses, including appropriate data visualization, will be conducted to determine the impact of specific stressors on mental health, linking responses over time to allow for the observation of changes in trends.

**Conclusions:**

The PSSI is an intuitive and evidence-informed tool that can aid postsecondary institutions in evaluating the sources of student stress on their campuses. This multisite project will make a substantial contribution to the current literature regarding postsecondary student stress and allow institutions across the country to improve the tailoring of upstream mental health services in order to directly support the unique needs of their student body. Opportunities for knowledge translation and exchange are discussed.

**International Registered Report Identifier (IRRID):**

DERR1-10.2196/27713

## Introduction

Over the past decade, the mental health of postsecondary students has increasingly been identified as a major public health concern. The majority of those attending postsecondary education in Canada belong to the 18-to-25-year age group, referred to as “emerging adulthood” [1]. As a result of increased autonomy, and a lack of permanent roles and responsibilities, emerging adults tend toward engaging in risk behaviors as maladaptive methods of coping with the stressors they commonly face in the postsecondary setting. Simultaneously, emerging adulthood encapsulates a period of substantial brain and psychosocial development, where individuals are at increased sensitivity to risk factors for the development of mental illnesses, including substance use and sleep disruption [2]. Excessive stress among postsecondary student populations has been linked to languishing mental health, which, in turn, is associated with a number of negative outcomes, including the development of mental illnesses [3,4], poor academic performance [5], dropout [6], burnout [7], and, in extreme cases, self-injury and suicidal ideation [5,8]. Canadian postsecondary students reported a substantial prevalence of both stress and common mental illnesses, such as anxiety and depression (formally diagnosed or self-reported through the use of screening tools), through the National College Health Assessment II (NCHA II) survey [9]. In 2019, over 60% of students who responded reported experiencing above-average stress within the past 12 months (on an adjectival five-point scale ranging from “no stress” to “tremendous stress”), while 69% reported “overwhelming anxiety” and 52% reported feeling “so depressed it was difficult to function” [10]. Notably, prevalence estimates for self-reported symptoms of anxiety and depression, stress, and help seeking for mental health–related problems significantly increased between the 2013 and 2019 iterations of the survey [11].

Although many postsecondary institutions have attempted to ameliorate mental health–related issues by increasing on-campus treatment options, few have developed effective upstream services and support, such as mental health promotion and mental illness prevention [12,13]. In fact, a survey-based review of mental health and well-being services offered on postsecondary campuses across Canada revealed that only 70% of the institutional representatives who responded believe that students are well informed about mental health issues and available services on campus [14]. Additionally, almost all representatives indicated that their campuses could benefit from expanding upstream services, such as mental health promotion and outreach activities [14]. Better targeting of the main stressors students face may improve existing mental health promotion and mental illness prevention activities, but the ability to do so hinges on an improved understanding of student-specific stress.

Previous instruments designed to assess postsecondary student stress demonstrate substantial measurement weaknesses. Few involved a diverse sample of students in the development process (eg, engaging students only in a particular year, level, or program of study [15-18]), while others are too narrow (eg, items based solely on the literature or with little consideration for student input [19]) or too broad (eg, including stress-related items not relevant to the postsecondary setting [20]). In other cases, the process of development has not been disclosed at all, making it difficult to judge the validity of the instrument [15]. Additionally, the majority of existing instruments demonstrate weak validity and reliability [16,18,21] or have not been psychometrically assessed [19]. Finally, the majority of these instruments assess only a single element of stress, either the severity or the frequency of occurrence but not both. As a result of these measurement weaknesses, holistic data on the sources of postsecondary student stress are currently lacking.

In response to this gap, the Post-Secondary Student Stressors Index (PSSI) was developed, which evaluates 46 stressors by both severity and frequency of occurrence across five domains of stress: academics, learning environment, campus culture, interpersonal, and personal stressors [22]. The purpose of this paper is to report the study protocol for a cross-Canada, multisite, longitudinal release of the PSSI that will evaluate the sources of postsecondary student stress on diverse campuses across Canada. An improved understanding of student stressors will enable postsecondary institutions to better align their upstream campus mental health services with the needs of their student population. The key objectives of the study include the following:

1. Confirm the validity of the PSSI by conducting an additional psychometric evaluation of the tool using a large, varied sample of students from across Canada.
2. Partner with postsecondary institutions across Canada to evaluate the sources of postsecondary student stress and develop recommendations regarding improved tailoring of upstream mental health services.
3. Determine whether patterns of stress exist across various demographic factors of interest (ie, region, sex, level of study, area of study) and over time.

## Methods

### Project Overview

The PSSI was developed and validated through extensive collaboration with diverse samples of students over a 2-year period. To develop a tool that was specific enough to evaluate stress and individual experiences within the social context of being a student yet broad enough to be holistically applicable to a varied student body (and therefore useful to a variety of postsecondary institutions), students who varied in age, gender, area, level, and year of study participated in the development and refinement of the tool. Per the *Standards for Educational and Psychological Testing* [23], the authors collected four types of evidence for validity throughout the development and validation of the tool using a three-phase mixed methods sequential exploratory study design. Overall, the PSSI demonstrated strong psychometric properties, including test–retest reliability, relationships to like constructs, and an internal structure consistent with the expectations for an index. The complete development and psychometric analysis of the PSSI is detailed elsewhere [24].

The cross-Canada, multisite release of the PSSI is a cross-sectional, repeated measures study. Data is collected via online surveys at multiple time points over the course of the academic year. Responses are linked via a unique identifier, facilitating longitudinal data analysis. In addition to the PSSI, the survey includes additional measures to facilitate a thorough analysis of the relationship between student stress and mental health. The survey consists of three sections: (1) sociodemographic characteristics, (2) stress [24,25], and (3) mental health measures evaluating psychological distress [26] and resiliency [27] (Textbox 1). In the wake of COVID-19, additional items were added to the stress section of the survey in 2020 to capture students’ experiences of stress specific to the pandemic, drawing on items developed by the American College Health Association [28] and the Mental Health Research Canada [29]. Qualtrics survey software is used as the electronic platform for the survey.

**Survey measures.**

**Section I: Sociodemographics**

- Age
- Sex
- Relationship/marital status
- Children (yes/no)
- Living arrangement
- Year of study
- Level of study (undergraduate/graduate/professional)
- Area of study
- Student status (part-time/full-time)
- International student status (international/domestic)
- First-generation student (yes/no)
- Grade point average

**Section II: Stress**

- Post-Secondary Student Stressors Index (PSSI)
- COVID-19-related stress scales
- Perceived Stress Scale (PSS-10)

**Section III: Mental Health Measures**

- Psychological Distress Scale (K10)
- Mental Health Diagnosis History
- Connor–Davidson Resiliency Scale (CD-RISC 10)

This multisite study received ethics clearance from the Queen’s University Health Sciences and Affiliated Teaching Hospitals Research Ethics Board (TRAQ #6029173).

### Procedure

#### Sampling and Participants

Recruitment of students at each participating site is flexible, allowing for each institution to determine its own desired sample size and method of recruitment. Options for recruitment used to date include (1) drawing a sample of students through the Office of Institutional Research and Planning (or equivalent), (2) sending a recruitment notice via institutional student email listservs, (3) posting the recruitment notice to an institution-based posting service for research, and (4) posting the recruitment notice through institution-based social media channels, such as those run by student unions. Regardless of the method of recruitment, an anonymous URL is made available to prospective participants. As incentive to participate in the 2020-2021 launch of the survey, students who completed the survey were offered an opportunity to enter a raffle for a chance to win one of several US $20-$50 gift cards. Students are eligible to submit one raffle entry for each completed survey at each time point of data collection. A similar incentive is planned to be used moving forward with future iterations of the study.

#### Survey Dissemination

Participants receive the letter of information (LOI) for the study and the URL to the online survey in the recruitment notice. The LOI communicates the participants’ rights with respect to withdrawals, refusal to answer questions, and data confidentiality. The first item on the online survey asks prospective participants to provide their free and informed consent after reading the LOI. Participants must provide consent in order to progress in the survey. The survey consists of 30 questions (some of which are multi-item questions) and takes about 20 minutes to complete.

#### Data Collection and Processing

This study is cross-sectional and longitudinal in nature, with multiple data collection time points facilitating the analysis of changes and trends in stressors over the course of the academic year. Data collection time points are intentionally selected to avoid periods of time where stress levels might be artificially skewed (eg, assessing stress levels at final exam time is likely to result in an overestimation of usual mean stress levels). As a result, slight differences may occur between participating institutions on the basis of the timing of exam periods, reading weeks, etc. Data will be collected between September and April, with analysis conducted during spring.

#### Risk Mitigation

Although the risk is low, to mitigate any feelings of elevated stress or emotional distress that participants may feel after completing the survey, contact information for Student Wellness Services at each participating site and national mental health crisis lines (eg, Good2Talk and Canada Crisis Services) are provided at the end of the survey.

### Statistical Analysis

Descriptive statistics (frequencies, measures of central tendency, and dispersion, where appropriate) will be calculated for all demographic variables to evaluate the nature of the sample.

To evaluate the validity of the PSSI, correlational analyses with like constructs will be conducted, in addition to exploratory factor analysis (EFA) and confirmatory factor analysis (CFA). Scores on the PSSI will be correlated with those on the Perceived Stress Scale [25,30], the Kessler Psychological Distress Scale [26], and the Connor–Davidson Resiliency Scale [27] in order to assess relationships to other variables as evidence for construct validity. Results of the EFA using the multisite data will be compared to those of the EFA using the pilot data. The CFA will be used to evaluate model fit and further assess the internal structure of the tool. As the PSSI is designed as an index, not a scale, there is no prior assumption for correlation between groups of stressors [31]. Further details on the analytical implications of this concerning the validation of the PSSI are detailed elsewhere [24].

**Figure 1:**
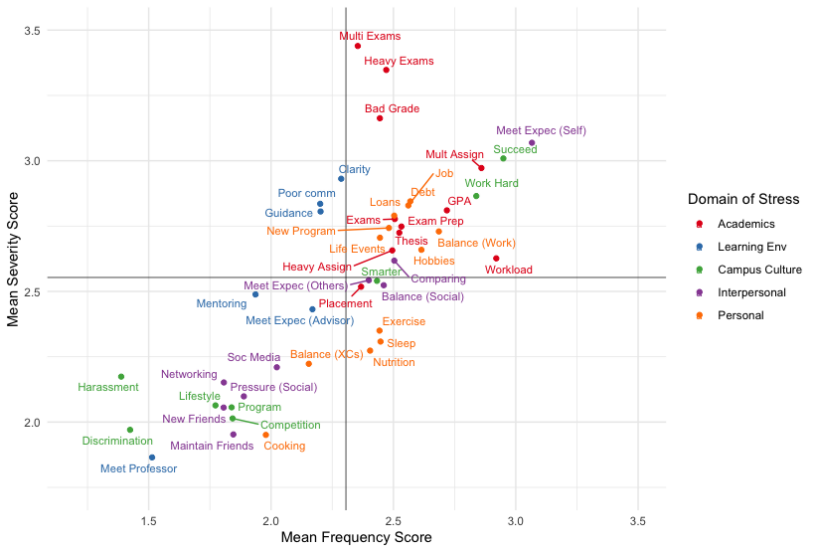
Post-Secondary Student Stressors Index (PSSI) stressors—all domains. Data (n=4954) are derived from the first timepoint of the 2020-2021 release of the PSSI. Comm: communication, Env: environment, Expec: expectations, GPA: grade point average, Heavy Assign: heavy assignment load, Mult Assign: multiple assignments, Multi Exams: multiple exams, XCs: extracurriculars.

**Figure 2:**
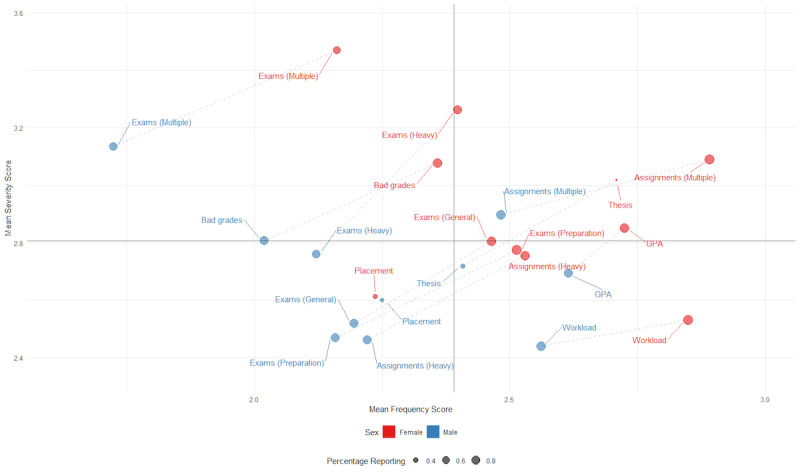
Academic stressors, stratified by sex. Data (n=535) are derived from the pilot test evaluation of the Post-Secondary Student Stressors Index [22]. GPA: grade point average.

Means for severity and frequency will be calculated for all stressor variables on the PSSI, with results plotted on a quadrant graph, color-coded by domain of stress (Figure 1). Results will also be plotted by individual domain of stress and can be stratified by demographics of interest, color-coded by demographic category (Figure 2). This depiction of stressors by mean severity (y-axis) and mean frequency (x-axis) will facilitate an intuitive understanding of the most severe and frequently occurring sources of stress (Figure 2, upper right-hand quadrant). Appropriate statistical tests will be conducted to determine whether the mean severity and frequency ratings for stressors are statistically significantly different. *T* tests for differences in means will be performed across demographic groups of interest, with corresponding 95% confidence intervals. Cumulative link mixed-effects regression analyses will be conducted to evaluate whether the mean severity for stressors has significantly changed over time (ie, over the course of the academic year across data collection time points) and across regions of Canada. Regression analyses will also be conducted to evaluate the impact of stressors on psychological distress. All statistical analyses will be conducted using R statistical software (R Foundation for Statistical Computing).

### Dissemination of Results

Following the completion of the study, institutional partners (coinvestigators) will get an opportunity to receive their own institution’s anonymized data for their own use. This data use agreement will create a positive community impact by allowing institutions to use the results of the PSSI for their campuses to inform improvements to their Student Wellness Services, facilitating knowledge exchange. Opportunities for knowledge translation will also be created. At the end of the survey, participants will be invited to add their names to an email list to receive a technical report of the findings of the study at the end of the study period. Several scholarly papers will be submitted to open access academic journals to ensure wide distribution of the study findings. Findings will also be disseminated through academic conferences via oral, poster, and workshop-style presentations. In addition, opportunities will be available for use of the anonymized data in student research and thesis projects.

## Results

**Figure 3:**
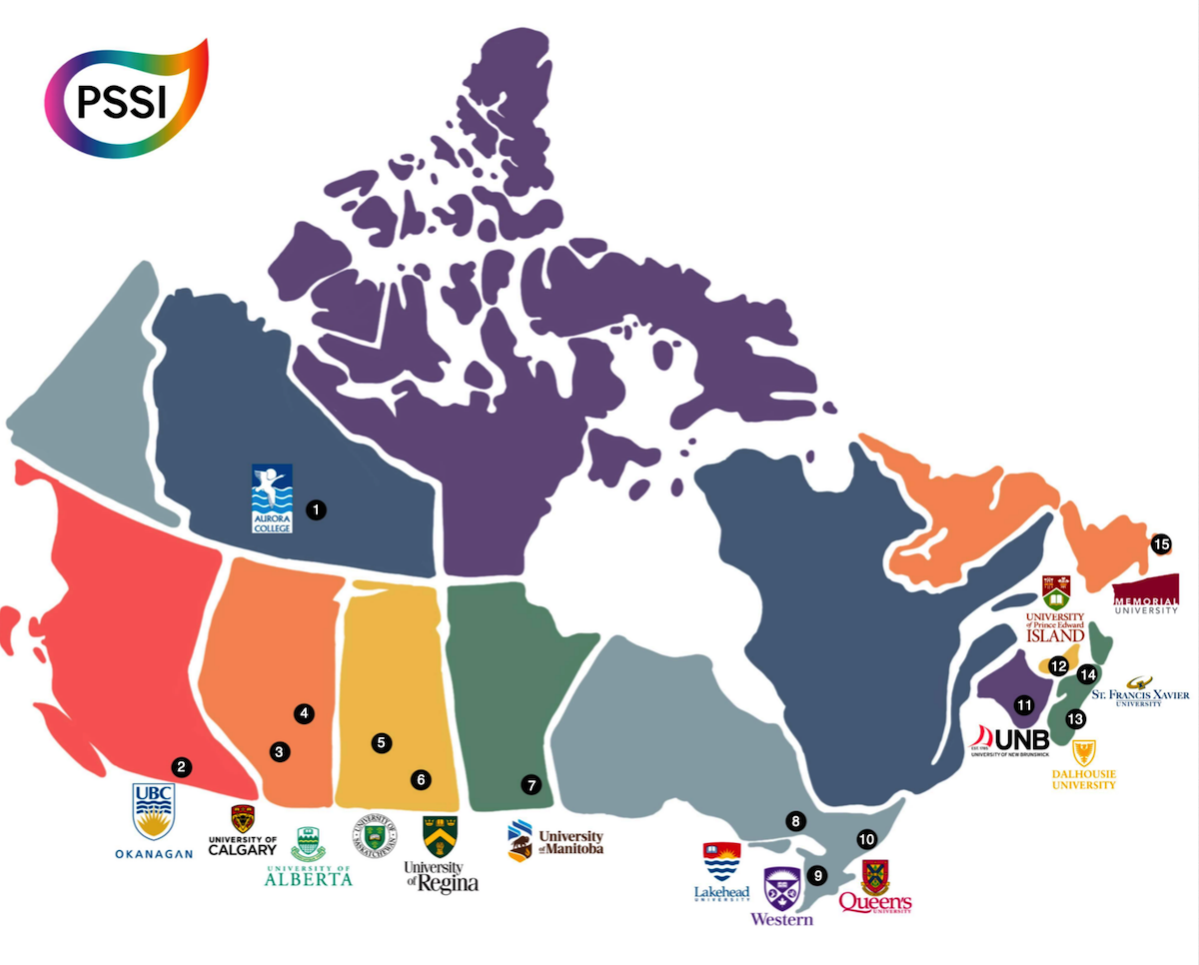
Participating institutions for project year 1 (2020-2021): (1) Aurora College (Northwest Territories); (2) University of British Columbia, Okanagan (British Columbia); (3) University of Calgary (Alberta); (4) University of Alberta (Alberta); (5) University of Saskatchewan (Saskatchewan); (6) University of Regina (Saskatchewan); (7) University of Manitoba (Manitoba); (8) Lakehead University (Ontario); (9) Western University (Ontario); (10) Queen’s University (Ontario); (11) University of New Brunswick (New Brunswick); (12) University of Prince Edward Island (Prince Edward Island); (13) Dalhousie University (Nova Scotia); (14) St. Francis Xavier University (Nova Scotia); and (15) Memorial University of Newfoundland (Newfoundland and Labrador). PSSI: Post-Secondary Student Stressors Index.

The goal of this research is to survey a wide variety of students enrolled at public postsecondary institutions across Canada. In 2020-2021, 15 universities were successfully recruited for the first year of the project, representing all but 1 province, in addition to 1 territory (Figure 3); this suggests that the institutional uptake of and response to the tool are positive. There are no inclusion or exclusion criteria for participation, aside from enrolment at a participating institution at the time of survey completion. Moving forward, additional institutions (including additional universities and colleges) will be added with each subsequent iteration of the survey.

## Discussion

### Aims and Strengths of the PSSI

Improved measurement of student stress will facilitate the improved targeting and alignment of mental health promotion and mental illness prevention services and support to best meet the needs of the student population. Enhancing upstream approaches targeting mental health promotion and stress reduction will help reduce not only the burden of mental health problems among the student population but also the demand currently placed on overburdened campus treatment services (eg, counseling). Available to Canadian postsecondary institutions at no cost in both English and French, the PSSI is an efficient and effective tool that provides a straightforward method of gathering data on the most severe and frequently occurring sources of stress on campus in order to support evidence-informed tailoring of campus mental health support. Implementation of the tool is flexible, with the ability to accommodate the timing of data collection in alignment with individual institution scheduling (exam periods, reading weeks, etc). The tool can also be used on its own or as an additional measure added to an existing or ongoing student mental health survey. Although the instrument’s current length may present a challenge in the latter option, a brief version of the PSSI is currently under development to facilitate easier addition onto existing survey tools.

The flexibility of this tool is a major strength. Given what we know about the impact of contextual factors and individual institutional cultures, it is not advisable to make blanket recommendations for student wellness services and mental health initiatives at the provincial or national level. Increasingly, governing bodies and leading organizations in this field (eg, the Mental Health Commission of Canada) are recommending the adoption of individualized, whole-campus approaches to student mental health and well-being that take into account the unique needs of each campus, using broad guidelines, such as those laid out in Canada’s Standard for Mental Health and Well-Being for Post-Secondary Students, as a guiding framework. The PSSI is an ideal tool for use alongside this framework; it provides institutions with an opportunity to obtain a clear road map of the most severe and frequently occurring stressors unique to their campuses and take appropriate, targeted action. As a result, gaining a representative sample of Canadian students to make generalizable recommendations is not the goal of the PSSI study. However, moving forward, making the best efforts to gain a representative sample of students at each participating institution will be a priority, as this will enable researchers to make recommendations that are more likely to have an impact on the mental health and well-being of the majority of students at an institution.

### Limitations

This research is subject to some practical and operational challenges. Many postsecondary institutions, particularly those that regularly participate in large-scale surveys, such as the NCHA II survey and, more recently, the Canadian Campus Well-Being Survey (CCWS) [32], are wary of oversurveying their students. Additionally, institutions that are already participating in these large-scale surveys feel they are already evaluating their students’ mental health, which can make it challenging to obtain buy-in.

While the NCHA II survey and the CCWS are valuable resources for broadly surveilling student mental health and well-being, the PSSI more specifically facilitates an in-depth analysis of potentially modifiable sources of student stress and is aimed at a tangible outcome: improving the tailoring of upstream mental health services and support to best serve students on campus. Additionally, when institutions opt for a repeated-measures longitudinal study design, the PSSI facilitates a more in-depth understanding of the timing of the need for services. Finally, our project team is open to working with institutions to avoid oversaturation of survey invitations by (1) finding survey time points throughout the academic year that do not conflict with other surveys and (2) selectively sampling students on the basis of a number of survey invitations received to date in the academic year.

Another challenge with respect to recruitment is related to the sharing of student information. Many institutions are restricted in their ability to share student contact information (eg, institutional emails) with outside investigators, while others have policies in place that restrict or prevent the recruitment of students via direct email contact. In cases like these, institutions should explore different approaches to recruiting, including (1) requesting an administrative staff member to send the recruitment notice to students via an institutional listserv or a newsletter; (2) posting through internal channels, such as campus notices and institutional research advertisement systems; and (3) reaching out to the student population through other channels, such as institutional-affiliated social media (eg, student unions). Although this flexibility in study design with respect to recruitment and sampling presents its own limitations, mainly with respect to determining representativeness of institutional samples, it is also a strength, in that it allows institutions with a variety of limitations associated with student access to participate in the project. However, the ability to make evidence-informed recommendations regarding the tailoring of campus mental health support is greatly improved by the institutions’ ability to secure a representative student sample.

### Conclusions

The PSSI is an intuitive and evidence-informed tool that can aid postsecondary institutions in evaluating the sources of student stress and tailoring upstream mental health services to directly support the unique needs of their campuses. The cross-Canada, multisite release of the PSSI is designed to be a long-term project, with the potential to observe whether improved tailoring of upstream services and support can produce changes in the patterns of student stress. Following each project year, additional institutions will be approached to participate in the project, with the goal of reaching as many postsecondary institutions across Canada as possible. To date, only universities have been engaged. Moving forward, we will also test the PSSI for efficacy among college populations to determine whether it needs an adaptation, acknowledging that potential item adjustment and additional validation work may be warranted among this unique population of students. A brief version of the PSSI is currently under development, in addition to an adaptation of the tool to explore its use as a student-facing self-assessment, with responses to the tool mapped to useful resources for students on the basis of stressors they find to be the most severe and frequently occurring. For example, if students find exam- and assignment-related stressors to be most severe, they might be directed to studying or time management resources the institution offers.

The PSSI is available upon request to institutions at no cost in both English and French.

## Abbreviations

CCWS: Canadian Campus Well-Being Survey
CFA: confirmatory factor analysis
EFA: exploratory factor analysis
GPA: grade point average
LOI: letter of information
NCHA II: National College Health Assessment II
PSSI: Post-Secondary Student Stressors Index
